# Heart failure and its progression is associated with changed lymphocyte profile in epicardial adipose tissue

**DOI:** 10.3389/fimmu.2026.1874851

**Published:** 2026-07-22

**Authors:** Barbora Judita Kasperova, Anna Cinkajzlova, Daniel Hlavacek, Jakub Mahrik, Ludek Horvath, Iveta Pleyerova, Barbora Dolezalova, Peter Ivak, Ivan Netuka, Vojtech Melenovsky, Sona Stemberkova Hubackova, Martin Haluzik, Milos Mraz

**Affiliations:** 1 Department of Diabetes, Diabetes Centre, Institute for Clinical and Experimental Medicine, Prague, Czechia; 2 First Faculty of Medicine, Charles University in Prague, Prague, Czechia; 3 Institute of Medical Biochemistry and Laboratory Diagnostics, First Faculty of Medicine, Charles University and General University Hospital, Prague, Czechia; 4 Cardiovascular Surgery Department, Institute for Clinical and Experimental Medicine, Prague, Czechia; 5 Third Faculty of Medicine, Charles University in Prague, Prague, Czechia; 6 Anaesthesiology and Resuscitation Department, Institute for Clinical and Experimental Medicine, Prague, Czechia; 7 Centre for Experimental Medicine, Institute for Clinical and Experimental Medicine, Prague, Czechia; 8 Cardiology Department, Institute for Clinical and Experimental Medicine, Prague, Czechia; 9 Metabolic Intensive Care Unit, Transplantation Centre, Institute for Clinical and Experimental Medicine, Prague, Czechia

**Keywords:** epicardial adipose tissue, heart failure, inflammation, lymphocytes, subcutaneous adipose tissue

## Abstract

**Introduction:**

Chronic inflammation is increasingly recognized as a key contributor to the development and progression of heart failure (HF), with epicardial adipose tissue (EAT) emerging as an important local immunomodulatory organ. This study examined lymphocyte populations in EAT and subcutaneous adipose tissue (SAT) across different stages of HF and compared them with individuals without HF to clarify their potential role in HF progression.

**Methods:**

Lymphocyte subsets in EAT, SAT, and peripheral blood were analyzed by flow cytometry in subjects with HF stage D, HF stage C, and subjects without HF. Circulating hormones and inflammatory proteins were quantified using ELISA and Luminex assays.

**Results:**

Subjects with HF stage D exhibited a reduction in T helper (Th), cytotoxic T (Tc), natural killer T (NKT), and B cells in EAT compared with both HF stage C subjects and subjects without HF, alongside changes observed in the circulation. In contrast, HF stage C was characterized by a significantly increased presence of Th2 and Th17 lymphocytes in EAT, indicating active immune remodeling during intermediate stages of HF. This stage was also associated with elevated circulating levels of Intracellular Adhesion Molecule-1, suggesting enhanced lymphocyte trafficking into adipose tissue. Across all study groups, EAT consistently contained a higher proportion of pro-inflammatory lymphocytes compared with SAT.

**Conclusion:**

Together, these findings demonstrate that HF progression is accompanied by dynamic and stage-dependent changes in lymphocyte composition within adipose tissue, supporting the concept that a progressively pro-inflammatory EAT microenvironment may contribute to myocardial inflammation dysfunction-associated HF progression.

## 1 Introduction

Heart failure (HF) is a multifaceted and chronic condition posing a significant global health challenge. It is characterized by impaired cardiac output, resulting in an inability to supply sufficient blood flow to meet the systemic metabolic demands, thus leading to symptoms such as dyspnea, fatigue, and fluid retention. Despite advances in medical research and treatment, HF remains a leading cause of morbidity and mortality worldwide. The prevalence of HF is estimated at 1-2% of the adult population and increases dramatically with age, affecting more than 10% of individuals older than 70 years (1). The complexity of HF stems from its diverse etiology, complex pathophysiology, and significant impact on patients’ quality of life (2), making both diagnosis and treatment challenging.

Multiple classification systems have been developed to define distinct clinical and pathophysiological subsets of HF. Functional classification using the New York Heart Association (NYHA) classification and characterization based on left ventricular ejection fraction are commonly used, while the American College of Cardiology/American Heart Association (ACC/AHA) classification incorporates symptomatic status with the presence of structural heart disease. A universal definition and classification proposed by the Heart Failure Society of America (HFSA), the Heart Failure Association of the European Society of Cardiology (HFA-ESC) and the Japanese Heart Failure Society (JHFS) based on guidelines described by ACC/AHA established four stages: Stage A (high risk of HF without structural heart disease or symptoms); Stage B (structural heart disease but without signs or symptoms of HF); Stage C (structural heart disease with prior or current symptoms of HF); and Stage D (refractory HF requiring specialized intervention) (3). Biomarkers play a critical role in HF diagnosis and risk stratification. Natriuretic peptides, such as B-type natriuretic peptide (BNP) and N-terminal pro-BNP (NT-proBNP), reflect cardiac wall stress and correlate with disease severity and prognosis (4). Additional biomarkers, such as troponins, indicate myocardial injury (5), while markers of inflammation (e.g., C-reactive protein) and fibrosis (e.g., galectin-3) provide complementary prognostic information (6).

Epicardial adipose tissue (EAT) is a unique fat compartment covering 80% of the heart’s surface and constituting 20% of total heart weight (7). EAT is metabolically active and exhibits both protective and pathogenic properties. Under physiological conditions, it serves as an energy reservoir, provides mechanical protection to coronary arteries, and secretes beneficial molecules that support cardiovascular function (8). Through vasocrine and paracrine signaling, EAT directly communicates with myocardium and provides free fatty acids for its energy metabolism (9). However, especially in the context of obesity, metabolic syndrome and HF, EAT undergoes pathological changes, leading to increased volume and altered, pro-inflammatory secretory profile. Enhanced inflammatory factor production creates a low-grade inflammatory microenvironment (10), exacerbates myocardial stress, promotes fibrosis, and impairs cardiac function. Additionally, EAT can directly infiltrate the myocardium (11), leading to mechanical and electrochemical disturbances and its proximity to coronary arteries implicates a potential role in the development of coronary artery disease, a common comorbidity of HF (12). Overall, these mechanisms underscore the potential relevance of EAT in HF pathophysiology. Deeper understanding of the role that EAT plays in HF may therefore open new therapeutic avenues.

The immune cell repertoire and their secreted products within adipose tissue are highly diverse, as different fat depots harbor distinct immune cell populations that dynamically respond to changes in systemic or local homeostasis. In lean adipose tissue, immune cells with anti-inflammatory properties, such as M2 macrophages, Th2 or T regulatory lymphocytes, predominate. In contrast, adipose tissue from subjects with obesity contains more immune cells with pro-inflammatory properties, such as M1 and metabolically activated macrophages, NK cells, B, Th1, Th17, and cytotoxic T cells, which infiltrate adipose tissue in response to stress signals, chemokines, and adipokines. Macrophages and lymphocytes subsequently serve as a significant source of pro-inflammatory cytokines (13–15). Similar immunological alterations have been observed in adipose tissue from subjects with cardiovascular diseases (including HF) (16). It has been described that EAT of subjects with HF exhibit pronounced immune activation, represented by accumulation of dendritic cells and T lymphocytes (both T helper and T cytotoxic cells), leading to increased production of pro-inflammatory IFN-γ cytokine (17). Inflammation within EAT has been recognized as a major contributor to the development of cardiogenic shock, as well as coronary artery disease (18, 19). However, the immune cell composition and subtype distribution within EAT across different stages of HF remains unclear.

In the present study, we focused on lymphocytes given their pivotal role as early responders that migrate into EAT during biological stress, initiating and shaping local immune responses (see graphical abstract). We characterized lymphocyte subpopulations and inflammatory profiles in peripheral blood, EAT and subcutaneous adipose tissue (SAT) from subjects across different stages of HF and compared them with subjects without HF. In addition, we measured circulating cardiometabolic and inflammatory markers to further elucidate the molecular mechanisms linking adipose tissue inflammation to HF progression.

## 2 Materials and methods

### 2.1 Study subjects

Study population consisted of 10 subjects with HF stage D, 46 subjects with HF stage C and 37 subjects without HF. The samples (EAT, SAT, blood) were obtained as a part of elective cardiac surgery. Inclusion criteria for patients with HF stage C consisted of HF history ≥ 6 months, left ventricular ejection fraction (LVEF) < 50%, brain natriuretic peptide (BNP) ≥ 693 ng/l and planned coronary artery bypass graft or valve surgery, while inclusion criteria for patients with HF stage D consisted of HF history ≥ 6 months, LVEF < 30%, BNP ≥ 693 ng/l, and planned implantation of mechanical circulatory support (LVAD – left ventricular assist device) or heart transplantation. For subjects without HF, inclusion criteria were absence of clinical signs of HF, LVEF > 50%, no significant valve disease, BNP < 150 ng/l, and elective cardiac surgery. Exclusion criteria for all patients included acute coronary syndrome, acute inflammatory state and active malignancy. Patients were primarily divided into groups according to objective parameters – echocardiography and biochemical markers of HF (LVEF, BNP levels) and HF requiring specialized intervention, while the subjective assessment of HF severity (NYHA classification) served as a secondary criterion (**Table 1**).

**Table 1:** Baseline characteristics in subjects with and without heart failure.

Baseline characteristics		HF stage D subjects	HF stage C subjects	Subjects without HF
Number (n; male/female)		10 (9/1)	46 (40/6)	37 (30/7)
NYHA stage (n)		IV (5)/III (3)	I-II (3)/II (20)/II-III (12)/III (11)/III-IV (1)	I (3)/I-II (6)/II (28)
Type of surgery (n, %)	CABG	0 (0.0)	24 (52.2)	24 (64.9)
	VLV^*^	0 (0.0)	13 (28.3)	11 (29.7)
	CABG+VLV	0 (0.0)	9 (19.6)	2 (5.4)
	HTx	5 (50.0)	0 (0.0)	0 (0.0)
	LVAD^x^	5 (50.0)	0 (0.0)	0 (0.0)
Age (year)		59.10 ± 3.23	64.97 ± 1.25	66.83 ± 1.38
Cachexia (n)		5/8 (62.5%)	3/46 (6.5%) ^D^	2/35 (5.7%) ^D^
Left ventricular ejection fraction (%)		21.50 ± 1.30	37.62 ± 1.34 ^D^	58.51 ± 1.00 ^D,C^
Brain natriuretic peptide (pg/ml)		1587.49 ± 492.16	354.81 ± 63.455	40.93 ± 5.342 ^D,C^
BMI (kg/m^2^)		26.58 ± 1.147	30.42 ± 0.807 ^D^	30.21 ± 1.096
Fasting glucose (mmol/l)		6.206 ± 0.598	6.828 ± 0.506	6.369 ± 0.318
HbA_1c_ (mmol/mol)		45.60 ± 3.631	51.13 ± 2.158	45.65 ± 1.533
Sodium (mmol/l)		137.2 ± 1.16	138.7 ± 0.35	138.9 ± 0.25
Potassium (mmol/l)		4.373 ± 0.156	4.136 ± 0.056	4.010 ± 0.054 ^D^
Chloride (mmol/l)		104.4 ± 1.925	104.9 ± 0.42	106.2 ± 0.41
Creatinine (µmol/l)		119.1 ± 19.37	85.95 ± 4.07	80.22 ± 3.30
Urea (mmol/l)		10.690 ± 1.139	6.939 ± 0.46 ^D^	5.754 ± 0.262 ^D^
Total bilirubin (µmol/l)		21.590 ± 3.119	12.220 ± 0.772 ^D^	16.427 ± 2.463
ALT (µkat/l)		0.538 ± 0.092	0.566 ± 0.038	0.589 ± 0.035
AST (µkat/l)		0.575 ± 0.141	0.472 ± 0.023	0.452 ± 0.030
ALP (µkat/l)		1.661 ± 0.235	1.233 ± 0.047	1.099 ± 0.050 ^D^
GGT (µkat/l)		2.119 ± 0.559	0.801 ± 0.107 ^D^	0.752 ± 0.107 ^D^
C reactive protein (mg/l)		12.920 ± 6.581	3.787 ± 0.475	3.119 ± 0.817 ^D^
Total cholesterol (mmol/l)		2.880 ± 0.185	4.033 ± 0.166 ^D^	3.576 ± 0.158
HDL cholesterol (mmol/l)		0.675 ± 0.062	0.986 ± 0.050 ^D^	0.946 ± 0.050 ^D^
LDL cholesterol (mmol/l)		1.630 ± 0.175	2.313 ± 0.141	1.930 ± 0.139
Non-HDL cholesterol (mmol/l)		2.106 ± 0.189	3.059 ± 0.165 ^D^	2.635 ± 0.138
Triglycerides (mmol/l)		1.276 ± 0.121	1.612 ± 0.120	1.537 ± 0.121
Apolipoprotein B (g/l)		0.671 ± 0.057	0.841 ± 0.046	0.756 ± 0.036
Total protein (g/l)		61.62 ± 2.621	75.90 ± 1.24	73.51 ± 1.14 ^D,C^
Albumin (g/l)		35.38 ± 1.98	45.91 ± 0.52 ^D^	45.70 ± 0.64 ^D^
Medication (n, %)	Anti-platelet therapy	1 (10)	30 (65.2) ^D^	26 (70.3) ^D^
	Anticoagulant	10 (100)	6 (13.0) ^D^	5 (13.5) ^D^
	β-blockers	9 (90)	35 (76.1)	28 (75.7)
	ACE-i/ARB	6 (60)	34 (73.9)	32 (86.5)
	ARNI	6 (60)	2 (4.4) ^D^	0 (0) ^D^
	MRA	9 (90)	25 (54.4) ^D^	2 (5.1) ^D,C^
	Statin	8 (80)	39 (84.8)	33 (89.2)
	Ezetimib	0 (0)	1 (2.2)	3 (8.1)
	SGLT-2i	4 (40)	2 (4.4)	0 (0)
	Digoxin	2 (20)	1 (2.2)	0 (0)
	Antiarrhythmics	5 (50)	4 (8.7)	1 (2.7)
	Loop diuretics	9 (90)	32 (69.6) ^D^	2 (5.4) ^D,C^

Values are Mean ± SEM. ^D^p<0.05 vs. HF stage D subjects; ^C^ p<0.05 vs. HF stage C subjects; One Way ANOVA/One Way ANOVA on Ranks. ^*^Includes VLV and VLV+AoR. ^x^Includes LVAD and VLV+LVAD. ACEi, Angiotensin-converting-enzyme inhibitor; AoR, Aortic surgery (including aortic replacement, stenting, or external aortic stenting) ALT, alanine aminotransferase; ARB, Angiotensin II Receptor Blocker; ARNI, Angiotensin Receptor Neprilysin Inhibitor; AST, aspartate aminotransferase; ALP, alkaline phosphatase; CABG, Coronary Artery Bypass Grafting; GGT, γ-glutamyltransferase; MRA, Mineralocorticoid Receptor Antagonist; NYHA, New York Heart Association; VLV, Valvular surgery.

All participants signed written informed consent prior to the enrolment into the study. Since the samples were taken as part of elective surgery, patients were not compensated for participation in the study. The study was approved by the Human Ethics Review Board, Institute for Clinical and Experimental Medicine (IKEM), Prague, Czech Republic (ethical approval code G-18-36) and was performed in agreement with the principles of WMA Declaration of Helsinki - Ethical Principles for Medical Research Involving Human Subjects.

### 2.2 Blood and tissue sampling

Blood samples were taken after overnight fasting and centrifuged for 10 min at 3000 x g within 30 min after withdrawal. Serum or plasma aliquots were subsequently stored at -80 °C.

The samples of EAT and SAT were taken perioperatively after 6–12 hours of fasting. All procedures were performed from median sternotomy, providing optimal access to the heart. The EAT samples were retrieved from the free wall of right ventricle, in order to avoid potential damage to underlying structures (especially right coronary artery). SAT samples were obtained from predetermined location in the lower pole of the sternotomy site. Freshly collected specimens in PBS buffer (0.01 M PBS, pH 7.4) were used for flow cytometry, and aliquots for further analyses were stored at -80 °C.

### 2.3 Hormonal and biochemical assays

Magnetic bead-based multiplex assay for the Luminex^®^ platform Milliplex^®^ Human Cardiovascular disease magnetic bead panel 4 (Merck KGaA, Darmstad, Germany) was used for detection of circulatory levels of soluble E-selectin (sensitivity 0.73 ng/ml), pentraxin 3 (sensitivity 0.06 ng/ml) and thrombomodulin (sensitivity 0.07 ng/ml) – only selected parameters were measured. ELISA kits were used for detection of Intercellular Adhesion Molecule-1 (ICAM1; sensitivity 2.2 ng/ml), galectin 3 (sensitivity 0.29 ng/ml), angiotensin converting enzyme 2 (ACE2; 40 pg/ml), leptin (sensitivity 0.2 ng/ml), all BioVendor R&D, Brno, Czech Republic, and adiponectin levels (sensitivity 0.2 ng/ml, Merck KGaA, Darmstad, Germany). The intra- and interassay variabilities for all assays were between 10.0 and 20.0%.

Biochemical parameter (blood glucose, glycated hemoglobin – HbA_1c_, sodium, potassium, chloride, urea, creatinine, total bilirubin, alanine aminotransferase (ALT), aspartate aminotransferase (AST), alkaline phosphatase (ALP), γ-glutamyltransferase (GGT), C reactive protein, total cholesterol, HDL cholesterol, LDL cholesterol, non-HDL cholesterol, triglycerides, apolipoprotein B, total protein, urine albumin, urine creatinine, non-esterified fatty acids, brain natriuretic peptide) and blood count analyses were measured at the Laboratory Methods Division, IKEM, Prague, Czech Republic by standard laboratory methods.

### 2.4 Isolation of stromal vascular fraction from adipose tissue and flow cytometry

The standard 0.5-1.0 g amount of adipose tissue was minced with sterile scissors, and visible blood vessels were removed. To minimize potential bias arising from differences in tissue size, all antibody staining reactions were normalized to the amount of tissue processed, and only samples with sufficient tissue yield (minimum 300 mg) were included in the analysis. Samples were washed in PBS, digested by 0.01% collagenase (Collagenase from Clostridium histolyticum; Sigma, St. Louis, MO, USA) for 30 min at 37 °C and centrifuged for 12 min at 1200 × g. Visible adipocyte fraction was then manually collected from the surface via a pipette with subsequent repeated washings and removal of remaining adipocytes from the supernatant. Finally, samples were filtered through Falcon^®^ 40 μm Cell Strainer (Becton, Dickinson and Company, Franklin Lakes, USA) to eliminate any remnant adipocytes from stromal vascular cells. Flow cytometry was performed using freshly isolated and filtered stromal vascular fraction or EDTA whole blood. A total amount of 100 μl of cell suspension with average 10^6^ cell content were labelled by fluorescently stained monoclonal antibodies (CD19 FITC/A07768, CD16 PE/A07766, CD56 PC5.5/B49189, CD4 APC/IM2468, CD8 AF700/B76279, CD3 A-AF750/A94680, CD45 KO/B36294, CD183 AF488/B68144, CD294 PE/A07413, CD196 PC7/B68132, all Beckman Coulter, Inc., Brea, CA, USA). The samples were labelled in the dark for 30 min at 2– 8 °C, and then red cells were lysed using Excellyse I (Exbio Prague, a.s., Vestec, Czech Republic) according to manufacturer’s instructions. Samples were analyzed on Navios Flow Cytometer (Beckman Coulter Inc., Brea, CA, USA). Data analysis was performed using FlowJo X 10.0.7r2 software (FlowJo, LCC, Ashland, USA). The minimal count of acquired events was 50,000.

The gating strategy for tissue and blood samples was as follows: debris, doublets and non-viable cells were excluded based on their characteristic forward- and side-scatter properties, lymphocytes were gated according to SSC properties and CD45 positivity, and then CD3- and CD3+ cells were assessed. B lymphocytes (CD19+CD3-CD45+ cells) and NK cells (CD16/56+CD3-CD45+ cells) were gated from CD3- cells. Th lymphocytes (CD4+CD3+CD45+ cells), Tc lymphocytes (CD8+CD3+CD45+ cells), and NKT cells (CD16/56+CD3+CD45+ cells) were gated from CD3+ cells. Th subpopulation were gated from Th lymphocytes: Th2 lymphocytes as CD294+CD183-CD196-CD4+CD3+CD45+ (CRTH2 positive) cells, Th1 lymphocytes as CD294-CD183+CD196-CD4+CD3+CD45+ (CXCR3 positive) cells, and Th17 lymphocytes as CD294-CD183-CD196+CD4+CD3+CD45+ (CCR6 positive) cells as published previously (19).

### 2.5 Statistical analysis

Statistical analysis was performed using SigmaPlot 13.0 (SPSS Inc., Chicago, IL, USA) and graphs were drawn using Microsoft Excel 2016 (16.0.5435.1000, Santa Rosa, CA, USA) and GraphPad Prism 10.1.0 (GraphPad software, Boston, MA, USA). Results are expressed as means ± standard error of the mean (SEM). Paired t-test/Wilcoxon Signed Rank Test, unpaired t-test/Mann-Whitney Rank Sum Test, One Way ANOVA/One Way ANOVA on Ranks, and Two Way ANOVA following by Holm-Sidak method were used for the assessment of intergroup differences, as appropriate. Pearson or Spearman correlation test was used to assess the association between biochemical and measured parameters and the backward stepwise selection method was performed to evaluate the influence of cachexia on different lymphocyte populations in EAT. Statistical significance was assigned to p ≤ 0.05.

## 3 Results

### 3.1 Baseline characteristics of study subjects

All groups of study subjects were the same age and gender ratio (**Table 1**). A part of study subjects was diagnosed with type 2 diabetes mellitus (T2DM) (4/10 – 40% in HF stage D group, 17/46 – 36.9% in HF stage C group and 12/37 – 32.6% in non-HF group; proportion of subjects with T2DM was not significantly different between groups – p=0.780).

Subjects with HF stage D had elevated levels of urea and creatinine, suggesting changes in renal function associated with HF as part of cardiorenal syndrome (20) (**Table 1**). Similarly, elevated levels of liver enzymes and total bilirubin in subjects with HF stage D can be related to passive congestion of the liver due to increased central venous pressure as a result of right-ventricular failure (21). Decrease in levels of total cholesterol, HDL cholesterol, non-HDL cholesterol, albumin and total protein in subjects with HF stage D reflects increased incidence of cachexia (evaluated as 5% body weight decrease in the last 6 months) in this group of patients (62.5% HF stage D subjects compared to 6.5% subjects with HF stage C or to 5.7% subjects from non-HF group), which is supported by lower BMI in these subjects (**Table 1**).

HF stage D group had increased lymphocyte and neutrophil peripheral blood count compared to non-HF group, which corresponds to higher measured CRP protein, likely due to the presence of low-grade inflammation (**Tables 1**, **2**). Similarly, neutrophil/lymphocyte ratio reflecting the level of systematic inflammation was higher in subjects from HF stage D group (**Table 2**). Decreased number of erythrocytes in subjects with HF stage D (**Table 2**) is in alignment with previously established connection between anemia and HF (22).

**Table 2:** Blood count analysis in subjects with and without heart failure.

Blood cell types	HF stage D subjects	HF stage C subjects	Subjects without HF
Leukocytes (10^9/l)	11.830 ± 1.191	8.689 ± 0.537	7.038 ± 0.469 ^D,C^
Neutrophils (10^9/l)	6.643 ± 0.828	5.183 ± 0.380	3.763 ± 0.231 ^D,C^
Lymphocytes (10^9/l)	0.932 ± 0.110	1.873 ± 0.101 ^D^	1.766 ± 0.0925 ^D^
Monocytes (10^9/l)	0.650 ± 0.108	0.668 ± 0.044	0.550 ± 0.029
Eosinophils (10^9/l)	0.241 ± 0.151	0.220 ± 0.020 ^D^	0.172 ± 0.017
Basophils (10^9/l)	0.034 ± 0.003	0.0428 ± 0.003	0.0362 ± 0.003
Erythrocytes (10^12/l)	3.347 ± 0.221	4.296 ± 0.076 ^D^	4.159 ± 0.115 ^D^
Neutrophil/lymphocyte ratio	7.981 ± 1.27	3.033 ± 0.23 ^D^	2.377 ± 0.225 ^D^

Values are Mean ± SEM. ^D^p<0.05 vs. HF stage D subjects; ^C^p<0.05 vs. HF stage subjects; One Way ANOVA/One Way ANOVA on Ranks.

Concerning the markers that have been associated with higher risk of HF progression, elevated levels of ICAM-1 have been observed in subjects with HF stage C compared to subjects without HF (**Table 3**), which corresponds with pro-inflammatory markers and leukocyte changes.

**Table 3:** Serum levels of selected heart failure markers in subjects with and without heart failure.

Heart failure markers	HF stage D subjects	HF stage C subjects	Subjects without HF
sE selectin (ng/ml)	x	16.664 ± 1.045	18.831 ± 1.193
Pentraxin 3 (ng/ml)	x	0.803 ± 0.067	0.821 ± 0.060
Thrombomodulin (ng/ml)	x	4.964 ± 0.186	4.857 ± 0.253
ICAM-1 (ng/ml)	x	433.14 ± 20.13	365.09 ± 19.06 ^C^
Galectin 3 (ng/ml)	x	11.532 ± 0.464	11.903 ± 0.637
ACE 2 (ng/ml)	x	6.407 ± 7.987	6.234 ± 1.016
Leptin (ng/ml)	x	17.252 ± 2.215	12.191 ± 1.643
Adiponectin (µg/ml)	x	8.824 ± 1.138	6.635 ± 0.574

Values are Mean ± SEM. ^D^p<0.05 vs. HF stage D subjects; ^C^p<0.05 vs. HF stage C subjects; unpaired t-test/Mann-Whitney Rank Sum Test; sE selectin, soluble E selectin; ICAM-1, intercellular adhesion molecule; ACE2, angiotensin converting enzyme 2.

### 3.2 Changes in distribution of immune cells in EAT and SAT and in peripheral blood

**Figure 1:**
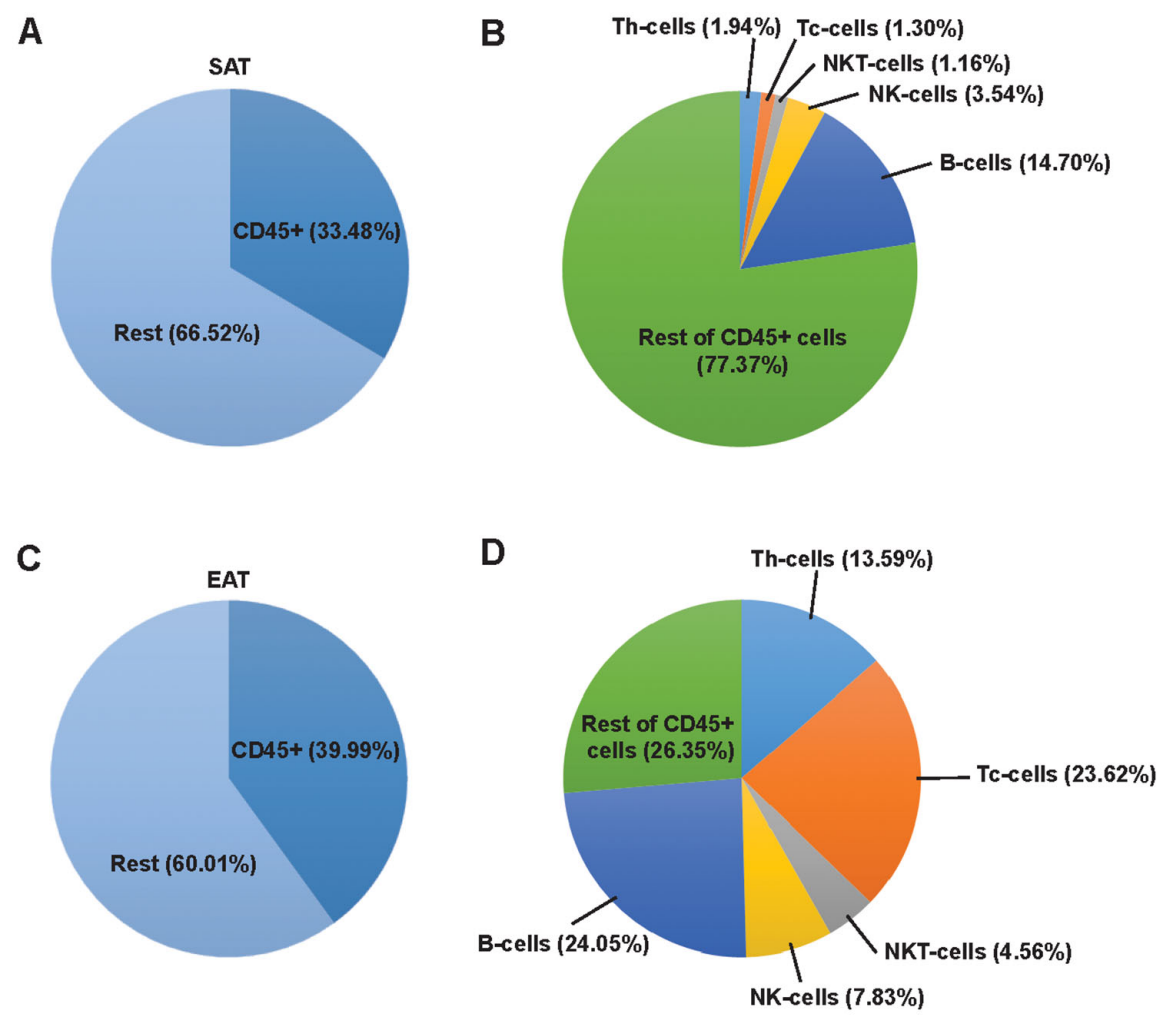
The difference in lymphocyte subpopulations between SAT **(A, B)** and EAT **(C, D)** in the whole study group; SAT in contrast to EAT comprised the same percentage of CD45+ cells with lower number of all assessed lymphocyte subpopulations with the exception of NK cells; EAT, epicardial adipose tissue; HF, heart failure; SAT, subcutaneous adipose tissue; Th cells, T helper lymphocytes; Tc cells, T cytotoxic lymphocytes; NK cells, Natural killer cells; NKT cells, Natural killer T cells.

There was no significant difference in total levels of infiltrated lymphocytes gated according to CD45+ positivity and SSC properties between EAT and SAT (p=0.093); however, there were increased populations of Th, Tc, NKT (all p<0.001) and B cells (p=0.001) in EAT compared to SAT (**Figure 1**).

**Figure 2:**
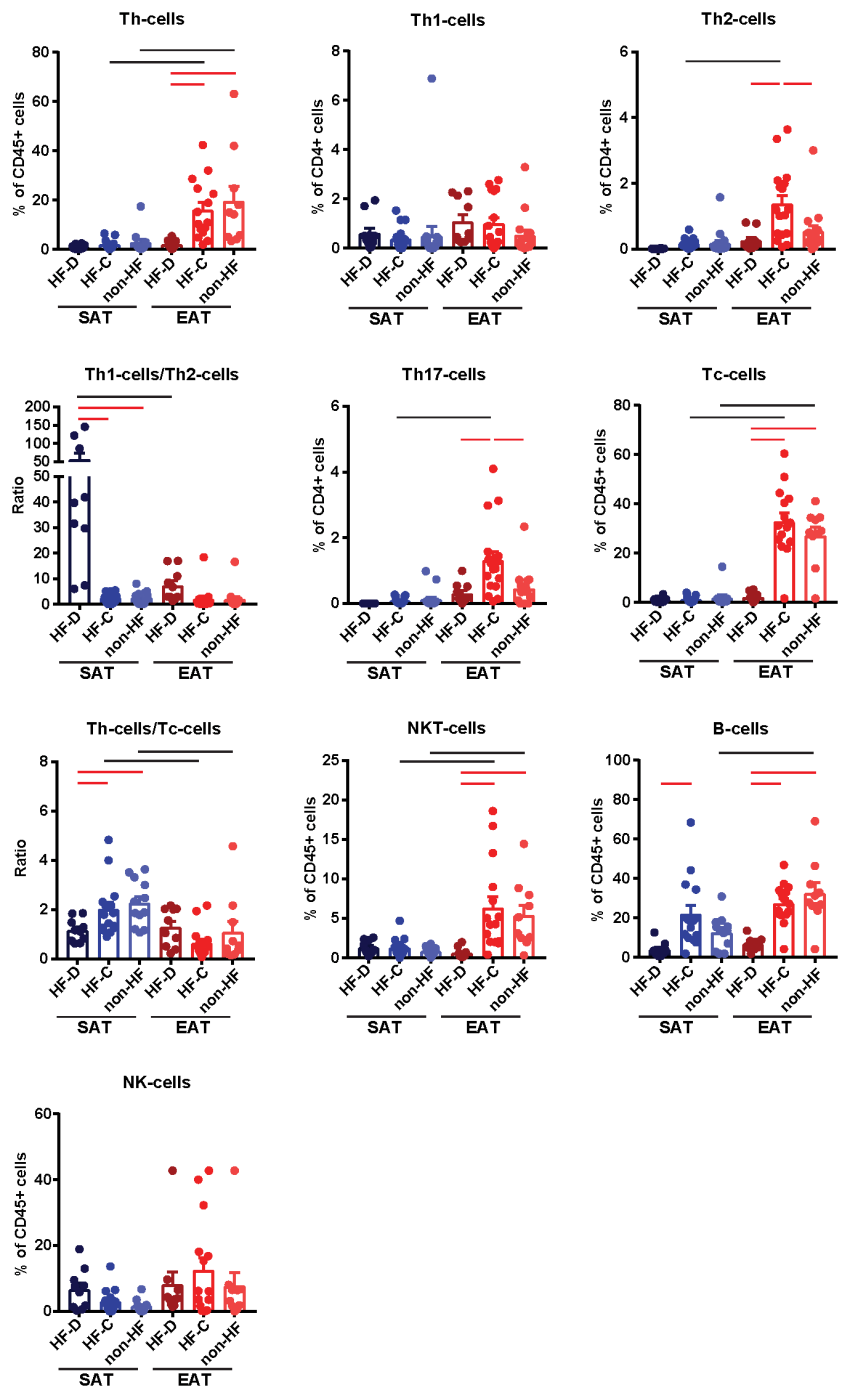
Percentage of lymphocyte subpopulations in subcutaneous and epicardial adipose tissue depending on heart failure stage. The figure displays differences in lymphocyte composition between SAT and EAT (black line) in subjects with different stages of HF (HF stage C, HF-C; HF stage D, HF-D) and without heart failure (non-HF) as well as between groups within single fat depot (red line). Values are Mean ± SEM. p<0.05, red line – difference between groups; black line – difference between subcutaneous and epicardial adipose tissue; Two Way ANOVA. EAT, epicardial adipose tissue; SAT, subcutaneous adipose tissue; Th cells, T helper lymphocytes; Tc cells, T cytotoxic lymphocytes; NK cells, Natural killer cells; NKT cells, Natural killer T cells; Results of Th cell subpopulations are expressed as percentage of CD4+ cells, results of Th, Tc, NKT, NK and B lymphocytes are expressed as percentage of CD45+ cells.

Analysis of EAT has shown an increase of CD45+ cells in HF stage D subjects compared to subjects from HF stage C and non-HF group (52.77 ± 2.87 vs. 39.22 ± 2.75 and 32.87 vs. 4.23%, p=0.001). However, the percentage of Th, Tc, NKT and B cells was lower in subjects from HF stage D group in comparison to subjects from HF stage C group and non-HF group. The highest percentage of anti-inflammatory Th2 cells and pro-inflammatory Th17 cells was present in EAT of HF stage C subjects (**Figure 2**). Even though the levels of pro-inflammatory Th1 cells were not significantly different between groups, their ratio to Th2 cells tended to be higher in subjects from HF stage D group, pointing to a more pronounced inflammation in their EAT.

Analysis of SAT has shown a decrease in the presence of CD45+ cells in subjects from HF stage D group compared to subjects from HF stage C and non-HF group (18.64 ± 3.66 vs. 42.86 ± 3.48 and 32.28 ± 2.95%, p<0.001). No change of Th, Tc, NK and NKT cells was found between groups, while the HF stage D subjects had lower percentage of, B cells in comparison to patients from HF stage C(**Figure 2**). As in the EAT, Th1/Th2 ratio in SAT was higher in patients with HF stage D.

When further comparing individual cell populations based on the stage of HF and type of adipose tissue, higher presence of Th2 cells and Th17 cells was detected in EAT compared to SAT in HF stage C group (**Figure 2**). The percentage of Th, Tc and NKT cells was higher in EAT of subjects from HF stage C and non-HF group. B cells percentage was increased in EAT of non-HF subjects compared to SAT.

Concerning peripheral blood, the percentage of CD45+ cells was significantly lower in subjects from HF stage D group compared to non-HF subjects (19.47 ± 3.76 vs. 39.05 ± 2.57%, p<0.001). Subjects with HF stage D have demonstrated a lower percentage of Th cells, Th2 cells and higher percentage of Th1 cells compared to HF stage C and non-HF groups, while the Tc cells percentage was decreased only in comparison with HF stage C group and B cells were decreased in comparison with non-HF subjects. Th1/Th2 ratio was increased in HF stage D and HF stage C groups compared to non-HF group (**Table 4**).

**Table 4:** Flow cytometry analysis of lymphocytes in peripheral blood .

shown as % of CD45+ cells	HF stage D subjects	HF stage C subjects	Subjects without HF
Th cells	4.161 ± 1.003	11.349 ± 0.862 ^D^	12.338 ± 1.192 ^D^
Th1 cells	0.810 ± 0.255	0.024 ± 0.007 ^D^	0.021 ± 0.006 ^D^
Th2 cells	0.0915 ± 0.0171	11.094 ± 0.848 ^D^	11.823 ± 1.167 ^D^
Th17 cells	0.762 ± 0.376	0.359 ± 0.083	0.384 ± 0.094
Tc cells	1.781 ± 0.517	4.271 ± 0.419 ^D^	4.571 ± 0.781
Th/Tc cells ratio (fold change)	2.559 ± 0.336	3.382 ± 0.553	3.505 ± 0.577
NKT cells	0.281 ± 0.0753	0.077 ± 0.009	0.072 ± 0.013
NK cells	3.713 ± 2.728	1.720 ± 0.417	3.498 ± 1.567
B-lymphocytes	0.991 ± 0.271	1.796 ± 0.236	1.967 ± 0.206 ^D^
Th1/Th2 cells ratio (fold change)	7.768 ± 2.383	0.031 ± 0.007	0.002 ± 0.001^D,C^

Values are Mean ± SEM ^D^ p<0.05 vs. HF stage D subjects; ^C^ p<0.05 vs. HF stage C subjects; One Way ANOVA/One Way ANOVA on Ranks. CD marker characteristic is stated in section Material and Methods. Th cells, T helper lymphocytes; Tc cells, T cytotoxic lymphocytes; NK cells, Natural killer cells; NKT cells, Natural killer T cells.

Associations of measured cell populations with systemic parameters are shown in **Table 5**. For the evaluation of cachexia influence on the lymphocyte population in EAT, we performed a multivariable analysis with single lymphocyte population in EAT as dependent variable, the degree of heart failure as independent parameter and cachexia as forced variable. The results shown, that the main T cell population – Th cells with p=0.002, Tc cells with p ≤ 0.001, NKT cells with p=0.020, and B cells with p ≤ 0.001 can be predicted from a linear combination of the degree of heart failure.

**Table 5:** Correlation analysis: single cell types with measured parameters.

Leukocyte types	Tissue	NYHA	BMI	Glycemia	Sodium	Potassium	Creatinine	Urea	Bilirubin	ALT	ALP	GGT	CRP	Cholesterol	HDL cholesterol	Non-HDL cholesterol	Triglycerides	Total protein	Albumin	Urine creatinine	BNP	E selectin	Pentraxin 3	Troponin T	Thrombomodulin	Adiponectin
Th	SAT																									
	EAT	-0.58							-0.37				-0.35	0.40				0.48	0.70							
	Blood	-0.41	0.32					-0.59	-0.48		-0.31	-0.36		0.42	0.41	0.29		0.37	0.64							
Th1	SAT	0.46							0.49				0.42													
	EAT						0.61	0.42	0.34			0.33	0.35							-0.41	0.45					
	Blood													-0.28					-0.63							
Th2	SAT	-0.33	0.32					-0.42										0.48	0.54			0.39				
	EAT																				0.34		0.42			
	Blood	-0.52	0.35				-0.43	-0.62	-0.43					0.41	0.50			0.44	0.66	0.33						0.39
Th17	SAT			-0.34														0.40	0.66							
	EAT			-0.37															0.48		0.43		0.39		0.42	
	Blood												0.37												-0.33	
Tc	SAT																	0.58								
	EAT	-0.55			0.36			-0.55	-0.38				-0.39	0.53					0.86						-0.51	
	Blood	-0.31							-0.29																	
NKT	SAT							0.34																		
	EAT	-0.45			0.40			-0.59	-0.41	0.38			-0.41	0.39				0.46	0.77							
	Blood					0.37												-0.47	-0.47	-0.38						
B	SAT	-0.37						-0.40	-0.41		-0.39	-0.41	-0.35	0.38	0.53				0.50							
	EAT	-0.68				-0.35		-0.35	-0.40					0.47	0.42			0.41	0.64							
	Blood	-0.32						-0.33	-0.28					0.34	0.31				0.37							
NK	SAT	0.53						0.47															0.53	0.54		
	EAT		-0.40														-0.36						0.48			
	Blood																									

Filled values are R results of correlation tests with p<0.05, results with p>0.05 are not shown. Results are coloured according to R strength: 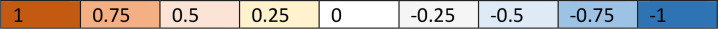

All measured parameters from **Tables 1**-**3** were used for analysis. If no significant results for particular parameter was determined, parameter was not integrated into the table.

## 4 Discussion

Adipose tissue immune cells are considered important players of low-grade inflammation, adversely influencing adjacent tissues and modulating their physiological functions. Thus, the immune cells that have infiltrated the EAT and are involved in direct interaction with myocardium seem to have an adverse impact on congestive HF (23), atrial fibrillation (24, 25) or coronary artery disease (19). This negative effect is exerted through various mediators, including adipokines, cytokines and oxidative stress molecules (26, 27). Here, we found an association between severity of HF and EAT lymphocyte changes, hinting towards a pro-inflammatory milieu in EAT and enhanced pro-inflammatory profile of subjects with HF.

Lymphocytes represent a substantial immune cell population whose expansion in adipose tissue could potentially be crucial in the development of local and systemic low-grade inflammation (14). Th1 and Th17 cells are associated with pro-inflammatory reactions and M1 macrophage activation, while Th1 cells have also been linked to increased prevalence of acute coronary artery syndrome and atherosclerosis (28). In this study, Th1 cell prevalence did not differ in EAT relative to SAT in any of the groups, whereas HF stage C subjects exhibited higher Th17 cell percentage in EAT compared to SAT. Overall increased expansion of Th1 and Th17 populations in EAT is consistent with previously described shifts in Th1/Th2 and Th17/Treg cell balance toward pro-inflammatory phenotype (29). However, our results do not support the conclusion that these balance shifts are related to the stage of HF, as the SAT and EAT of subjects with HF stage D contained a comparable number of Th1 cells, while also showing decreased percentage of Th17 cells in comparison to HF stage C in epicardial adipose tissue depot.

In contrast to Th1 and Th17 cells, Th2 cells reduce the risk of myocardial infarction and stroke (30, 31). Th2 cells associated with anti-inflammatory reactions predominate in healthy lean subjects (32, 33). We found a higher percentage of Th2 cells in EAT of subjects with HF stage C. However, Th1/Th2 ratio was significantly higher in HF stage D group in SAT with a similar trend in EAT, thus underlining the pronounced inflammatory response, which was further confirmed by markedly increased Th1/Th2 proportion also in their peripheral blood. Even though the expansion of Th2 cell population in EAT of HF stage C subjects would indicate positive anti-inflammatory effects, the fibrogenic effect of Th2 cell cytokines IL-13 and IL-4 enhancing synthesis of the extracellular matrix proteins during chronic tissue repair process might, conversely, have a worsening effect on HF progression (34, 35). Moreover, IL-4 potentially produced by Th2 cells/M2 macrophages in EAT could contribute to the accumulation of mast cells, which infiltrate the EAT in response to coronary artery disease (36) and whose population was found to be increased in subjects with chronic HF (37). Nevertheless, Th2 cell infiltration in EAT seems to be limited, as subjects with HF stage D have similar values as patients without HF, although these results could be influenced by the general decrease in Th cell population.

HF progression significantly disrupts T cell content in peripheral blood with a reduction in Th cell population and a shift towards a more pro-inflammatory phenotype with increased Th1 and Th17 and reduced Th2 cells. Patients with HF stage D often exhibit features of premature cellular senescence, characterized by reduced thymic output of naïve T cells and accumulation of dysfunctional memory T cells, ultimately decreasing the effective Th cell population (38). Elevated ICAM-1 levels may reflect this process, as its expression is upregulated by senescence-associated molecules such as p53 (39), while also influencing T lymphocyte infiltration into EAT and local inflammatory milieu (40). Th cells in HF stage D patients may undergo increased rate of apoptosis due to high levels of oxidative stress and inflammatory mediators (41). Additionally, chronic antigen exposure can lead to T cell exhaustion, characterized by reduced proliferation and function of Th cells. Severe HF state (HF stage D) may further impair hematopoiesis through bone marrow dysfunction, reducing lymphocyte production, including Th cells (42).

T cytotoxic (CD8+) cells are another cell population potentially adversely influencing HF progression as they have been proposed to accumulate in adipose tissue and increase inflammation by pro-inflammatory cytokine production (43, 44), as also described in ischemic heart disease (45, 46). Furthermore, Tc cells can negatively influence myocardium by granzyme B production leading to cardiomyocyte apoptosis, adverse post-ischemic cardiac remodelling (47) and cardiac fibrosis (48). As described previously (17), we found positive correlation between Tc and Th cells infiltration into SAT as well as in EAT (R = 0.918, resp. R = 0.694, both p<0.001). Interestingly, both non-HF and HF stage C subjects have shown lower amount of Tc and Th cells in SAT compared to EAT, while HF stage D subjects have the relatively low content of Tc and Th in both adipose tissue depots. In addition, HF stage D subjects have higher Th/Tc cell ratio compared to HF stage C subjects in EAT.

NK and NKT cells represent innate lymphocyte populations with pleiotropic roles in the development of cardiovascular diseases. While both cell types can promote inflammatory responses through cytokine production, they have also been shown to exert regulatory effects that limit adverse cardiac remodeling and fibrosis (49–51). Reduced NK cell numbers have been reported in circulation and EAT of subjects with coronary artery disease (19, 50), while experimental studies suggest cardioprotective roles of both NK and NKT cells (51). In our study, NK cell amount did not differ between groups in either adipose tissue depot, while NKT cells were reduced in EAT of HF stage D subjects. Together, these findings suggest innate lymphocyte-mediated protective mechanisms may be more prominent during earlier stages of HF and become progressively impaired with disease progression.

Although an anti-inflammatory B cell subpopulation has also been described (52), current evidence largely supports a pathogenic role of B cells in HF through antibody and cytokine production (53, 54), and immune cell recruitment (55). The levels of both IgG3 and the C3c part of complement in the myocardium correlate with duration and severity of HF (53). Surprisingly, in contradiction to previously published studies showing increased infiltration of B cells in EAT compared to SAT (19, 52, 56), we found the higher percentage of B cells in EAT compared to SAT present only in non-HF subjects, but not in HF stage C and D subjects.

An additional explanation for the reduced proportions of T, B, and NKT lymphocytes observed in EAT of HF stage D subjects may also involve the expansion of other immune cell populations. Consistent with the increased proportion of CD45+ cells observed in these subjects, previous studies have demonstrated immune cell accumulation in inflamed adipose tissue or myocardium and in advanced HF (53, 56). Therefore, the observed findings likely represent both lymphocyte-specific changes and broader remodeling of the immune cell infiltrate. Interestingly, transcriptomic analysis by Zhang et al., 2023 (17) identified lymphocyte activation as a prominent feature of EAT in HF patients, highlighting the complexity of immune remodeling within this tissue.

All these changes in the lymphocyte profile can be also associated with the development of cachexia, especially in subjects with HF stage D. Although these subjects had normal BMI, the diagnosis of cachexia was confirmed as they have shown signs of body weight loss (>5% in the last 6 months), inflammation, worsened liver function (increased ALP and GGT levels) anaemia, and lymphopenia (57, 58). Moreover, BMI may be falsely elevated due to fluid retention associated with cardiac and renal dysfunction. Cachexia itself is associated with systemic inflammation, cardiac dysfunction (59) and profound adipose tissue remodeling, including increased lipolysis, impaired adipogenesis, enhanced thermogenesis and altered immune responses (60). As shown in rat cancer cachectic models, cachexia is associated with a significant increase in macrophage population within white adipose tissue under enhanced secretion of TNF-α and MCP-1 expression by adipocytes and/or pre-adipocytes (61). The potential role of cachexia in the observed alterations of lymphocyte populations during HF progression is supported by several strong correlations between specific lymphocyte subsets and cachexia-related markers, such as urea, albumin, and cholesterol levels. On the other hand, our multivariable analysis with cachexia as a forced variable factor confirmed the dependence of Th, Tc, NKT and B cell subpopulations on the degree of HF. In addition, malnutrition or micronutrient deficiencies common in advanced HF may additionally impair lymphocyte function (62). Also, medicaments commonly used in the management of HF stage D, such as beta-blockers, ACE inhibitors, and mineralocorticoid receptor antagonists, may contribute to altered adipose tissue immune composition through their immunomodulatory effects. For instance, SGLT-2i were more frequently used in patients with HF stage D, and previous data demonstrated that these agents exert anti-inflammatory effects within EAT, including reduced macrophage infiltration and lower prevalence of Th1 and Th2 lymphocytes (63). The interpretation of our findings should also take into account the differences in surgical indication between study groups as patients with HF stage D underwent LVAD implantation or heart transplantation, while subjects with HF stage C and controls underwent CABG or valve surgery. These distinct clinical settings may be associated with differences in myocardial remodeling, systemic inflammation, and immune activation that could affect lymphocyte trafficking and adipose tissue immune composition. Nevertheless, all samples were obtained during elective procedures using a uniform sampling protocol, and the observed immune alterations followed a pattern consistent with HF severity, supporting the notion that disease progression represents an important driver of the detected changes.

Taken together, progression of HF is linked to changes in lymphocyte populations within EAT, which may lead to enhanced production of pro-inflammatory mediators (*e.g.* cytokines, adipokines, fibrokines) that have a proven negative effect on myocardium. In our study, we have confirmed that adipose tissue differs in immune cell composition based on localization, as EAT has been shown to contain larger population of T and B lymphocytes compared to SAT, while SAT in turn contains a larger population of macrophages, dendritic cells, neutrophils and other cells expressing CD45 antigen. However, while our findings indicate substantial changes in lymphocyte composition during HF progression, they should be interpreted within the context of the immune populations analyzed. EAT contains a diverse repertoire of immune cells, including macrophages, dendritic cells, neutrophils, and innate lymphoid cells, which are also known to contribute to adipose tissue inflammation and remodeling. Therefore, the observed changes should be regarded as evidence of lymphocyte-specific immune remodeling.

## 5 Conclusion

HF is a complex clinical syndrome closely linked to chronic low-grade inflammation. In our study, we have shown that progression form HF stage C to HF stage D is associated with significant changes in lymphocyte composition within both SAT and EAT. We have also demonstrated that the effect of HF severity is not uniform across adipose tissue depots, as the transition from HF stage C to HF stage D appears to influence SAT and EAT differently. Together, these findings suggest that lymphocyte-specific immune remodeling of EAT accompanies HF progression and may contribute to the inflammatory milieu associated with myocardial dysfunction and disease progression. Further studies incorporating a broader characterization of immune-cell populations will be necessary to fully define the role of EAT immune cell remodeling in HF pathophysiology and its potential as a therapeutic target.

## 6 Limitations

Several limitations of this study should be acknowledged. First, the number of subjects in the study cohorts was relatively limited, particularly in the HF stage D group. Nevertheless, the cohort size reflects the severity and rarity of advanced HF requiring LVAD implantation or heart transplantation and provided sufficient statistical power for the analyses performed. Second, the study groups differed in the type of surgical procedures performed. Subjects with HF stage D underwent LVAD implantation or heart transplantation, whereas subjects with stage C and non-HF controls underwent CABG and/or valvular surgery. Although EAT samples were collected according to a standardized protocol before any major surgical manipulation, differences in conditions may have contributed to local and systemic immune activation. Therefore, some of the observed differences in lymphocyte composition may reflect factors related to the surgical setting in addition to HF severity itself. Furthermore, EAT samples were obtained exclusively from the free wall of the right ventricle. Thus, our findings primarily reflect the lymphocyte characteristics of this specific EAT depot and may not fully represent the immunological (lymphocyte) profile of EAT in other locations. Finally, pharmacological treatment differed between groups and may have influenced the observed EAT lymphocyte-specific phenotype, particularly in subjects with advanced HF. Notably, a substantial proportion of subject with HF stage D received SGLT-2 inhibitors, which have been reported to attenuate inflammation and immune-cell infiltration in EAT. Because medication use was closely linked to HF severity and the sample size was limited, the effects of individual therapies could not be reliably distinguished from those of disease progression.

## Data Availability

The raw data supporting the conclusions of this article will be made available by the authors, without undue reservation.
